# Learning-based cable coupling effect modeling for robotic manipulation of heavy industrial cables

**DOI:** 10.1038/s41598-022-09643-6

**Published:** 2022-04-11

**Authors:** Fangli Mou, Bin Wang, Dan Wu

**Affiliations:** State Key Laboratory of Tribology, Department of Mechanical Engineering, Tsinghua University, Beijing, China

**Keywords:** Mechanical engineering, Engineering

## Abstract

The robotic manipulation of a heavy industrial cable is challenging to model and control because of the high number of degrees of freedom and the rigid-flexible coupling dynamics. In this paper, we report the development of modeling the cable effect and control methodology for robotic cable manipulation. Our cable effect model is based on the 2D convolutional neural network, which is a deep learning-based method uses the effective cable representation method to achieve the accurate, generalizable, and efficient estimation of the cable coupling forces and torques. Practical problems such as the measurement limits and time efficiency are considered in our method for real applications. With these approaches, we are the first to solve the problem of dynamic payload effect caused by heavy industrial cables in experimental cases. The used control methodology combines the active disturbance rejection control framework with the sliding mode control method, which can acquire promising tracking performance. We integrate our cable effect model into the control scheme, and demonstrate it satisfies the high-quality robotic manipulation of heavy cables. The performance of the proposed method is assessed with both a simulated system and real robot system. The results show that our method can estimate the cable coupling effect with over 85% accuracy and accomplish manipulation with a positioning error less than 0.01 mm. This reveals that our method is promising for robotic manipulation of heavy industrial cables and can accomplish the challenging cable insertion task.

## Introduction

Manipulating heavy cable objects with robots has a wide range of industrial applications and creates tremendous benefit. In the current assembly of an aircraft, workers need to manually lay and mount over 200 km of aeronautic cables^1^, which not only limits the production efficiency but also brings huge strain on the workers. With the development of aviation and manufacture technology, the mission of industrial cable assembly will increasingly face a number of complex and diverse manipulation tasks.

**Figure 1:**
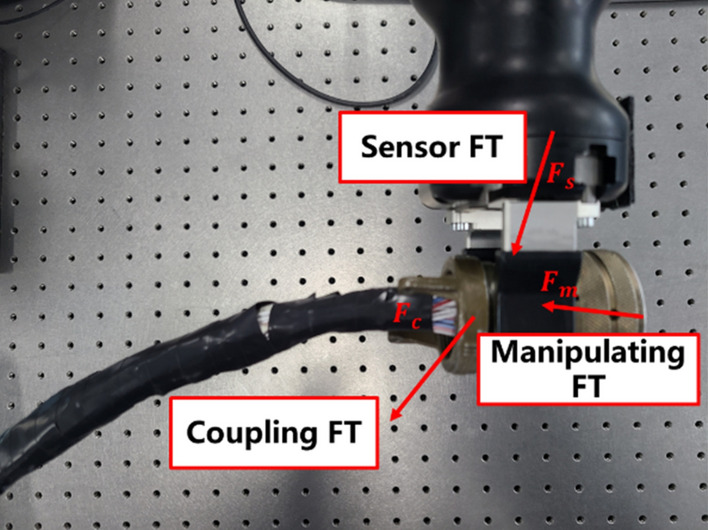
The task of robotic aeronautic cable insertion manipulation.

Different from manipulating the rigid objects, the complex dynamics and high dimensionality of deformable objects makes the manipulation tasks much more challenging in robotics. For this reason, relative studies mainly focus on solving the motion planning problem for manipulating light and soft deformable objects^2–4^. In these works, the deformable objects were assumed to have no influence on the robot system and the using robot was regarded with the perfect ability to manipulate the deformable payload as what is preplanned. However, when manipulating heavy and stiff objects such as aeronautic and industrial cables, the payload effect is rather obvious and not appropriate to be simply ignored. As shown in Fig. 1, to accomplish the robotic industrial cable insertion manipulation task, we need to guarantee high positioning accuracy and decouple the cable effect to control the contact and insertion process.

Cable objects can be categorized as deformable linear objects (DLOs), modeling DLO dynamics in 3D space is challenging due to its high dimensions and complex dynamics. Generally, analytic modeling techniques can be divided into mesh-based methods and meshless methods^5^. The mesh-based models are more commonly used compared to meshless methods, and can be categorized into continuum models and discrete models according to the consistency of the mesh. The discrete models are mainly represented as Mass-Spring-Damper (MSD) models, which represent DLO bodies using a set of mass particles connected by springs in a lattice structure. The finite element methods (FEM) are one of the most popular continuum methods^7^, where the DLO is discretized using a set of discrete geometric parts called finite elements, and partial differential equations (PDEs) provided by continuum mechanics need to be solved. These methods are used for computing the deformation of DLOs, the applied forces and torques are the input of models and the time efficiency is less important. Hence, these conventional modeling methods are not suitable for modeling the real-time cable effect that we concern in robotic cable manipulation. Recently, data-driven models have been widely used due to their ability to directly learn from observations and have the potential of being accurate and computationally light enough for use in robot control and planning^8^. And several works have been focused for modeling deformable bodies, fluids and DLOs^9–12^. However, similar to those analytic physics-based models, these data-driven methods are also aimed for cable planning in the simulation. Problems such as measurement ability and computing efficiency are not concerned, which makes these methods unsuitable in real robot control applications.

Several works have been focused on the specific problem of manipulating DLOs^13–19^. The task of tying a knot using the robot is discussed in^20^, the method based on robots manipulating at high speed and the assumption that the dynamic behavior of the DLO can be obtained from algebraic calculations of the robot motion. Wang et al.^21^ addressed the tight-tolerance insertion tasks for string and rope using the approximate Jacobian in conjunction with a virtual magnetic field emanating from the hole and a re-grasping method for reducing the approaching error. Liu^17^ investigated the untangling ropes problem for robots using an RGB-D sensor to perceive the knot structure. In these studies, the manipulated DLOs are light and soft with low elasticity, which makes the manipulated object can be approximated as a geometrical model. Moreover, these tasks are represented as a proof of concept and the precision of the robot manipulation does not need to be considered much. Correspondingly, manipulating a DLO into a desired shape is a common action in automotive industry. A robotized wire handling system was developed for assembling the wrapped cables in a car^22^. The wrapped cables cannot be simply treated as the geometrical objects, which makes the average speed of assembly have to be slow in order to reduce the coupling effect caused by the cables. Based on programming by demonstration, Rambow et al.^23^ presented a method using the admittance control framework to overcome the uncertainties for robotic deformable object manipulation. It is noteworthy that current studies mentioned above mainly focused on planning a path for robots manipulating DLOs under the assumption that the manipulated DLOs cause no influence to the robot. However, it is not appropriate to ignore the cable dynamics when manipulating heavy and stiff cables such as aeronautical and industrial cables, especially when the task needs high manipulation precision such as the cable insertion task shown in Fig. 1. A method for heavy cable manipulation was proposed in^24^; however, this method is rather primary and not practical for real applications.

This paper addresses the robotic manipulation tasks having precision requirement for heavy and stiff cables. We present a practical and effective method for modeling the cable coupling effect and controlling a robot to achieve the heavy cables manipulation with high precision. Our core idea is to design and use the representative features-based cable representation to provide an estimation of the payload effect and embed this learned model into an active disturbance rejection control (ADRC)^25^ based sliding mode controller (SMC). A convolutional neural network (CNN) is used for modeling the complex cable effect, the proposed method is considered and designed from a practical viewpoint, on account of the engineering background. We demonstrate the effectiveness of our method for the positioning task of cable manipulation, and quantitatively show that the proposed method is significantly more effective in accomplishing the robotic manipulation of heavy cables compared to the existing method.

Succinctly, the key contributions of this paper are as follows:

- As far as the authors are concerned, this is the first attempt to consider and solve the problem of dynamic payload effect caused by manipulating heavy DLOs in experimental cases. We propose an efficient cable representation method without requiring expensive measuring costs, which reduces the dimensions of original features by 69%.
- We propose a learning-based cable coupling effect modeling method using a residual learning neural network, which shows improved generalization compared to the published modeling method.
- We demonstrate our method in both simulated and real environments. Quantitative comparisons show that our method can estimate the coupling forces and torques with over 85% accuracy, reduces prediction errors by an average of 67% and reduces the positioning error to less than 0.01 mm compared to the baseline, respectively.

The rest of this study is organized as follows. Section “Preliminaries” introduces the problem definition and the limits using the existing approaches; section “Methods” provides a description of our method for modeling the cable effect in manipulation; simulation and experimental results are demonstrated and discussed in section “Results and Discussions”; conclusions are drawn and future work is discussed in section “Conclusions”.

## Preliminaries

In this section, we briefly describe the coupling effect caused by cable in the robotic manipulation, we also show the common techniques for cable modeling and our previous study, which address the motivation of our work in this paper.

### Cable effect in manipulation

**Figure 2:**
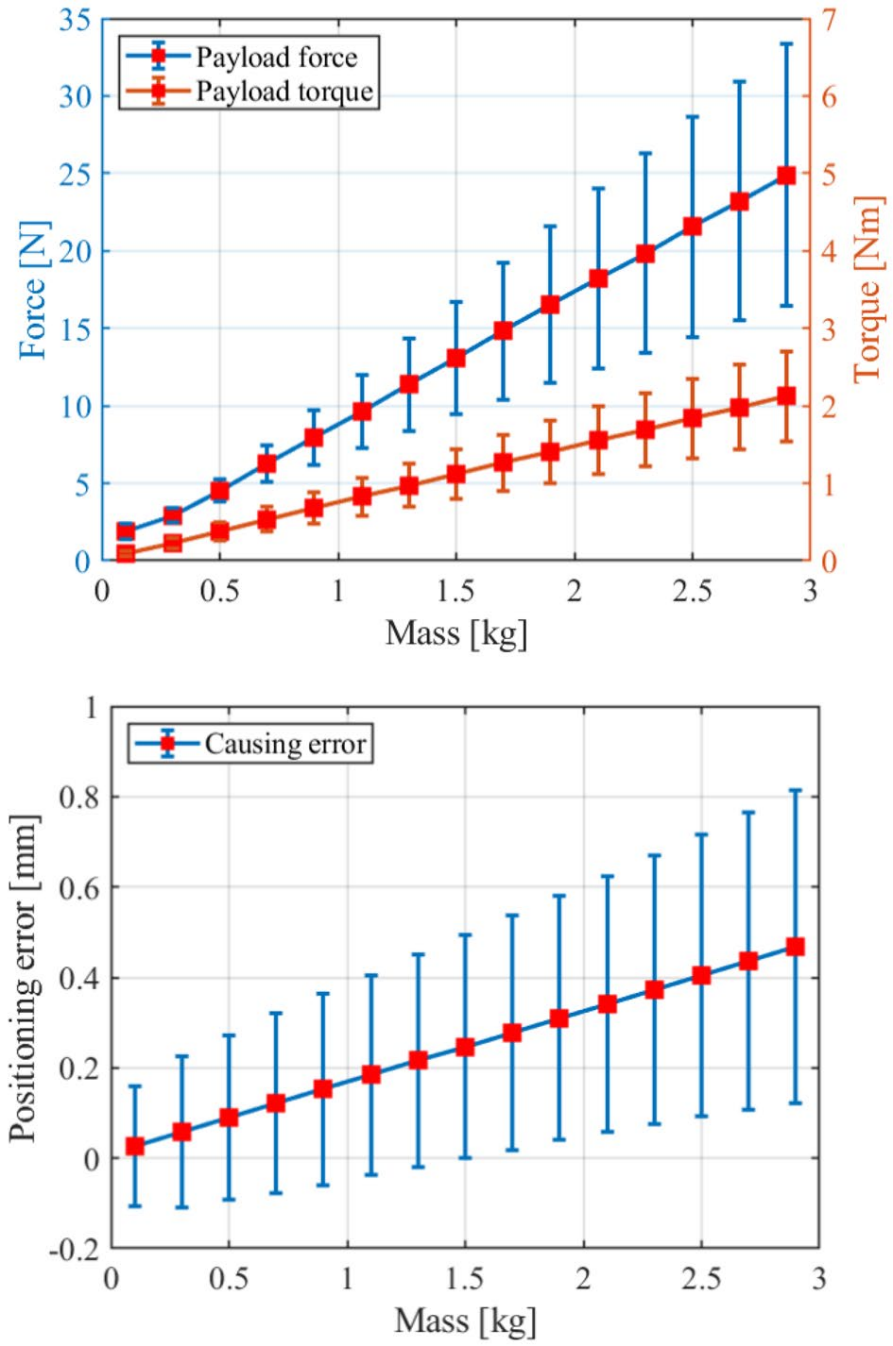
The illustration of cable effect and corresponding influence to robot with different cable properties.

Same as the rigid payload, the cable object brings coupling forces and torques to the robot in manipulation tasks as shown in Fig. 1. We define this coupling forces and torques (as ${\mathbf{F}}_{c}$ in Fig. 1) as the cable effect in this paper. However, due to the deformable characteristic, commonly used payload identification method for rigid payload^26^ which estimates the inertia parameters using the joint states of robot cannot be applied to the cable cases. Figure 2 shows the cable effect and positioning error of different cables manipulated by a UR 5e robot in the simulated environment (detailed in section “Results and Discussions”). We should note that Fig. 2 shows a general phenomenon, robots with different rated payload have similar performance when the cable weight has the same percentage of rated payload. As we can see in Fig. 2, a cable with larger weight and stiffness causes more significant cable effect. We also note that whether a cable is considered heavy or not in applications is relative to the applied robot and the requirement of the manipulation task. And in our experience, when the cable can cause effect compared to more than 10% of the robot’s rated payload, the coupling effect of the cable should be considered in the system design.

### Dynamic cable modeling methods

The conventional cable modeling methods are used to compute the cable deformation based on their current states and an input force, which are not suitable for modeling the cable effect here. To obtain the cable effect that we concern in robotic cable manipulation, we can use these mesh-based modeling methods to formulate the cable dynamics. If we can have every state of the elements of the mesh, we can achieve the cable effect by solving the equations. This is the basic idea of our work. In our previous work^24^, we use the features-based MSD method to simulate the cable effect, where we apply the partial least squares regression (PLSR) algorithm to perform feature points selection. However, this method has two main limits: First, the linear MSD model is less accurate and realistic, which makes the obtained model become a local model and the accuracy of estimation can be hardly improved. Second, the feature number of the final built model is too much (usually 32 feature points need to be measured for a 1 m cable in order to preserve a satisfactory accuracy), which makes the method rather impractical for applications.

The cable effect model can be directly used in the following three applications: (1) Decoupling the measured forces and torques, which makes it is possible to obtain and analyze the manipulating forces and torques (as shown in Fig. 1) without using extra sensors or equipment. (2) The cable effect model can be used in the controller design, which can improve the system performance and manipulation quality. (3) The cable effect model can provide a convenient and realistic dynamic model in the simulated environment, which can narrow the simulation-to-reality gap and make the simulated results more reasonable. In order to the accomplish these applications, the accuracy of estimation, cable representation method and the time efficiency of the cable effect model need to be addressed and investigated.

The material presented here is an improved and extended version of the preliminary study presented in^24^, both regarding the methodology and the quality of the results. In this paper, we mainly focus on three aspects of modeling the cable effect: the accuracy of estimation, the effective representation of the manipulated cable and the time efficiency of computing, which are crucial for accomplishing a robotic cable manipulation task in real applications.

## Methods

We formulate the problem of cable effect modeling as predicting the coupling forces and torques caused by manipulation using the measured cable states. In order to acquire a sufficiently accurate and concise model for robot control and manipulation, we first extract and construct features to describe the cable states (section “Representation of a cable”). Based on these features, we use a CNN to learn the cable effect model, and the structure of this network is described in section “Learning-based cable effect modeling”. Details on the applications of the proposed method are described in section “Application details”.

### Assumptions

The developments of the methods described in this paper are based on the following assumptions:

- The robot consists of rigid bodies, and knotting and tensioning of the cable do not happen during the task.
- The motion states of all the feature points of the cable are well known.

### Representation of a cable

**Figure 3:**
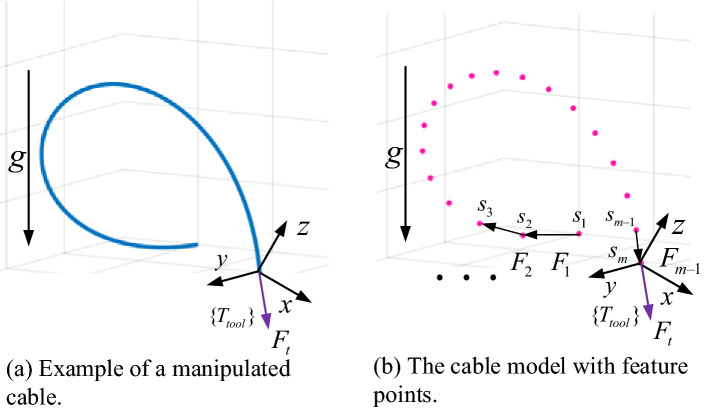
The feature model representation of the manipulated cable coupling effect to the robot.

Our cable representation is based on a nonlinear MSD model^6^. For a given manipulated cable form a manipulating point like shown in Fig. 3a, the coupling effect $F_{t}$ can be approximated using the internal forces $\{ F_{i} \}$ with feature points $\{ s_{i} \}$, as seen in Fig. 3b. We note that the gravity vectors are omitted for better illustration in these figures. For a heavy and stiff industrial cable, the axial stiffness is much larger and twisting usually needs to be avoided in manipulation. In addition, the shape of the cable is the most direct observation from images. Thus, we use the positions and velocities of the feature points as the original cable representation. We should note that our method cannot model the coupling effect caused by the twist of the cable, which is mainly reflected in the coupling torques and is less important in our task. Theoretically, we can have a more accurate model if we can use the twist information in modeling.

Using more feature points will make the obtained effect model have higher accuracy. However, for real applications, the number of feature points should be limited due to real-time hardware requirements. We now present a general scheme to acquire the robust feature points. Our optimization process uses the local effect model proposed in^24^. First, we execute robotic cable manipulation and collect the measurements, then segment the process data and perform feature points selection using the partial least squares regression (PLSR) algorithm for each segment. Next, the cable is divided into two segments based on the middle point of the cable and the statistics is performed, we calculate the average percentages of selected feature points in these two segments. Finally, we can extract and configure the feature points when given the desired number of feature points. Algorithm 1 summarizes the whole feature points configuration algorithm in pseudocode.
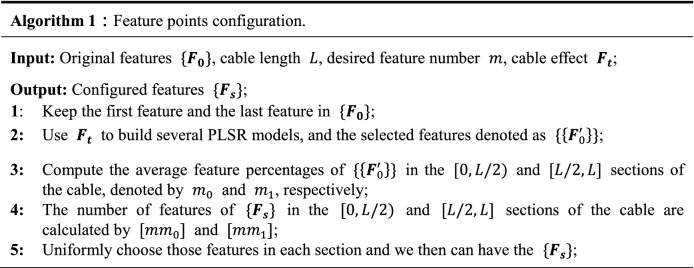


After choosing the feature points, we can define the original cable representation (OF) as

1 $${\mathbf{F}}_{OF} = [{\mathbf{p}}_{1} ,{\mathbf{v}}_{1} ,{\mathbf{p}}_{2} ,{\mathbf{v}}_{2} ,...,{\mathbf{p}}_{m} ,{\mathbf{v}}_{m} ] \in {\mathbb{R}}^{6m \times 1}$$

where ${\mathbf{p}}_{i} \in {\mathbb{R}}^{3 \times 1}$ and ${\mathbf{v}}_{i} \in {\mathbb{R}}^{3 \times 1}$ are the position and velocity of the feature points in the world frame, respectively, and $m$ is the number of configured feature points.

The original cable representation is not considered for modeling since ${\mathbf{F}}_{OF}$ depends on the establishment of the coordinate system, which has a poor ability for generalization. Furthermore, the relative cable representation (RF) can be defined as

2 $${\mathbf{F}}_{RF} = [{\mathbf{p}}_{2} - {\mathbf{p}}_{1} ,{\mathbf{v}}_{2} - {\mathbf{v}}_{1} ,...,{\mathbf{p}}_{m} - {\mathbf{p}}_{m - 1} ,{\mathbf{v}}_{m} - {\mathbf{v}}_{m - 1} ]$$

**Figure 4:**
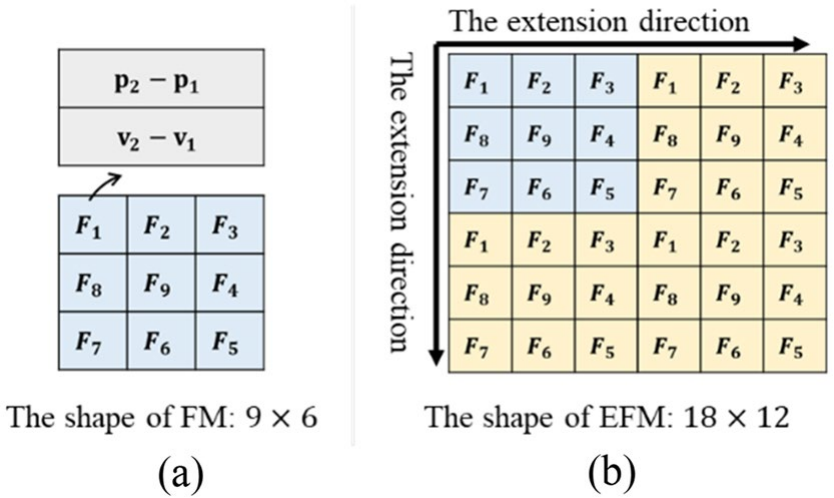
The FM (a) and EFM (b) representation of a cable.

The CNN method is widely used in solving feature extraction problems in machine learning and usually has better performance than simple fully connected network such as multilayer perceptron (MLP). Since a cable has the diminishing rigidity property^27^, we can recode the relative cable representation to realize centralization. We define this representation as the feature map of cable (FM), the FM is constructed using a helix method. The construction process of the FM with 10 feature points is shown in Fig. 4a. It should be noted that the FM representation is suitable for $m > 5$, and zero padding is processed from the front of the features when $m$ cannot be properly factorized. For better feature extraction, we can adopt the FM extension to strengthen the relation of feature points. We call this cable representation the extended feature map (EFM), which is shown in Fig. 4b. Note that the extension features can be the same data or the sequential data of the FM, and usually using the sequential data can have better performance in a dynamic state.

### Learning-based cable effect modeling

**Figure 5:**
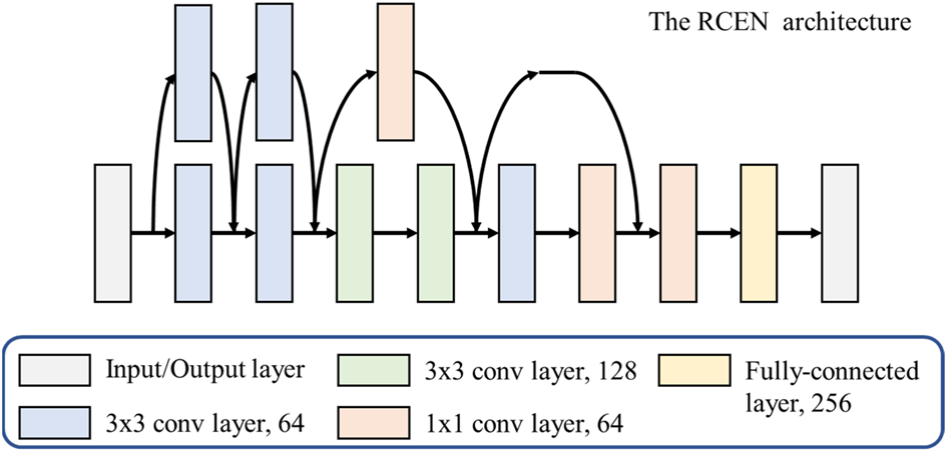
The network architecture for RCEN with 11 parameter layers.

To exploit this structure, we use an CNN with a residual learning framework^28^ to estimate the cable effect, as visualized in Fig. 5, we call this network the residual cable effect network (RCEN). The input of this network is the EFM representation of cable, the network output is the $6 \times 1$ scaling effect forces and torques vector as $\hat{y} = [\vec{F},a\vec{T}]$, where $a$ depends on the cable material and can be chosen based on the measurement data as $\sum {\left\| {\vec{F}} \right\|_{2} } /\sum {\left\| {\vec{T}} \right\|_{2} }$. The convolutional layers mostly have $3 \times 3$ filters. The network ends with a 256-dense fully-connected layer and a 6-dense fully-connected output layer. The activation function used is the exponential linear unit (ELU)^29^, except for the output layer. We adopt batch normalization and do not use dropout^30^, following the practice in^31^. Batch normalization, data augmentation and early stopping are the main technologies used in this paper to reduce the overfitting. We add Batch Normalization to each hidden layer of the network which is considered more suitable for training the convolutional layers and generate extra 10% of data points using the same noise level of measurement for training. We construct a loss function for combining the mean squared error and cosine similarity in order to obtain a prediction whose direction is relatively close to the measurement, to better analyze the coupling effect property in further research.

3 $$Loss = MSE(y,\hat{y}) + kCOS(y,\hat{y})$$

### Application details

The above approach is developed based on the fact that motion states of all the feature points of a cable can be completely measurable. However, a real system can hardly satisfy this requirement due to the camera ability and environment occlusion, resulting in some feature states that may be missing in the manipulation. To solve this problem, some state estimation methods^32–34^ for DLOs can be utilized. These estimation methods are usually complex and learning-based. Specific to our system, most states of the feature points can be observed in one measurement. Here, we present a rapid interpolation method to approximate the missing feature states in the measurement.

4 $$\begin{gathered} {\mathbf{p}}_{i} (t) = (1 - \mu )B({\mathbf{p}}_{o} (t),{\mathbf{p}}_{n} (t)) + \mu {\mathbf{p}}_{i} (t_{o} ) \hfill \\ {\mathbf{v}}_{i} (t) = ({\mathbf{p}}_{i} (t) - {\mathbf{p}}_{i} (t - \Delta t))/\Delta t \hfill \\ \end{gathered}$$

where ${\mathbf{p}}_{i} (t),{\mathbf{v}}_{i} (t)$ is the missing state of the feature point $s_{i}$, $B$ is the quadratic Bézier function, ${\mathbf{p}}_{o} (t)$ is the nearest feature point that can be measured before $s_{i}$, ${\mathbf{p}}_{n} (t)$ is the nearest feature point that can be measured after $s_{i}$, $\mu \in [0,1][0,1]$ is the weighted factor which can be chosen as a decaying exponential function of $t - t_{o}$, and ${\mathbf{p}}_{i} (t_{o} )$ is the last measurement state of feature point $s_{i}$. Here, the tangent vector of cable is used in constructing the Bézier function.

Now, we can use the proposed model to predict the coupling effect for a certain cable manipulation with a fixed manipulation length. Next, we normalize our method to apply different manipulation lengths. Let ${\mathbf{x}}$ be the nodal positions. The governing dynamic equations of a cable can be given using the FEM. And for each finite element, we have

5 $$\rho {\mathbf{\ddot{x}}} = \nabla \cdot \sigma + {\mathbf{f}}$$

where $\rho$ is the density of the material and the divergence operator turns the stress tensor back into a vector representing the internal force resulting from a deformed infinitesimal volume.

Considering a cable with manipulation length $L_{0}$ as $\zeta_{0}$, we can construct the effect model using the above approach as $H_{0}$. When the manipulation length of cable is changed to $L_{1}$, note that $L_{1} = \lambda L_{0}$ and the cable as $\zeta_{1}$, we can have a corresponding proportionate representation with $\zeta_{0}$. Let ${\mathbf{p}}_{\zeta 0} = {\text{L}}_{0} (t)$ and ${\mathbf{p}}_{\zeta 1} = {\text{L}}_{1} (t)$ for the parametric equations for $\zeta_{0}$ and $\zeta_{1}$, where $t \in [0,1]$. Then, for $\forall t \in [0,1]$, the equation ${\mathbf{p}}_{\zeta 1} = \lambda {\mathbf{p}}_{\zeta 0}$ is satisfied. Next, we discretize $\zeta_{0}$ and $\zeta_{1}$ with the same number of nodes, which means the finite element discretized depends on the parameter $t$. Since ${\mathbf{p}}_{\zeta 1} = \lambda {\mathbf{p}}_{\zeta 0}$, we have ${\mathbf{f}}_{\zeta 1} = \lambda {\mathbf{f}}_{\zeta 0}$ in (5). This result implies that we can use the model $H_{0}$ to compute the coupling effect of $\zeta_{1}$ by the configuring feature points with the same pattern and using the proportionate features $\lambda {\mathbf{p}}_{i}$ and $\lambda {\mathbf{v}}_{i}$.

### Consent to publish

The authors declare that they consent to publish this article.

## Results and discussions

We evaluate each of the components described in the above section and demonstrate them both in simulations and on real robots. We model a robot and cable system in the MATLAB/Simulink simulation environment. The robot model is UR 5e, which is the same as we used in the real experiments. The dynamic cable model is set up using the SimMechanics toolbox; one side of the cable is mounted on the ground and the other side is mounted on the end of the robot (i.e., held by the robot).

### Simulated verification

We generate data from different robotic manipulation trajectories in simulation. To obtain random cable deformation states with relatively apparent discrepancies, we use a chaotic sequence^35^ to generate robot trajectories with a setting region. In the simulation, a cable is modeled by 50 segments, where every two neighboring segments are connected by a special 6D spring-damper element. The cable parameters length $L$, radius $R$, and mass $m$ are set to 1.1 m, 7.54 mm and 1.5 kg, respectively. We generate about 1,200,000 data with different noise levels (detailed further) for further verification, and we measure the feature points of cable at 20 Hz in our method.

We first demonstrate the effectiveness of our cable representation method described in section “Representation of a cable”. The data are split into training and validation sets with an 80–20 split. We demonstrate the training and evaluation loss for each cable representation method in Table 1. The loss function is as described in (3), which approximates to the mean squared error for $\vec{F}$ and $10\vec{T}$ after training. For example, for cable representation with OF for all features, the presented model performance in Table 1 represents the average force prediction error is about 3.48 N and the average torque prediction error is about 0.348 Nm in the training set. Here, the number of total feature points is 51, and the number of configured features is 10. The training model is the same CNN with same training parameters. From these results between different methods in Table 1, we can know that the RF representation is better for learning the cable effect model and our feature configuration method can greatly decrease the feature points needed for learning, which reduces the computational cost and the measurement requirement of the system, making the physical system much more practicable and simpler. Besides, we can also see that the proposed FM and EFM representation can have better performance for training the cable effect model than directly using the RF representation.

**Table 1:** Model performance of different cable representation.

Cable representation	Model performance	
	Train	Test
OF for all features	12.1249	19.1262
RF for all features	12.2217	17.2607
RF for configured features	13.3554	17.7963
FM for configured features	12.0523	17.0985
EFM for configured features	11.3252	16.1384

**Figure 6:**
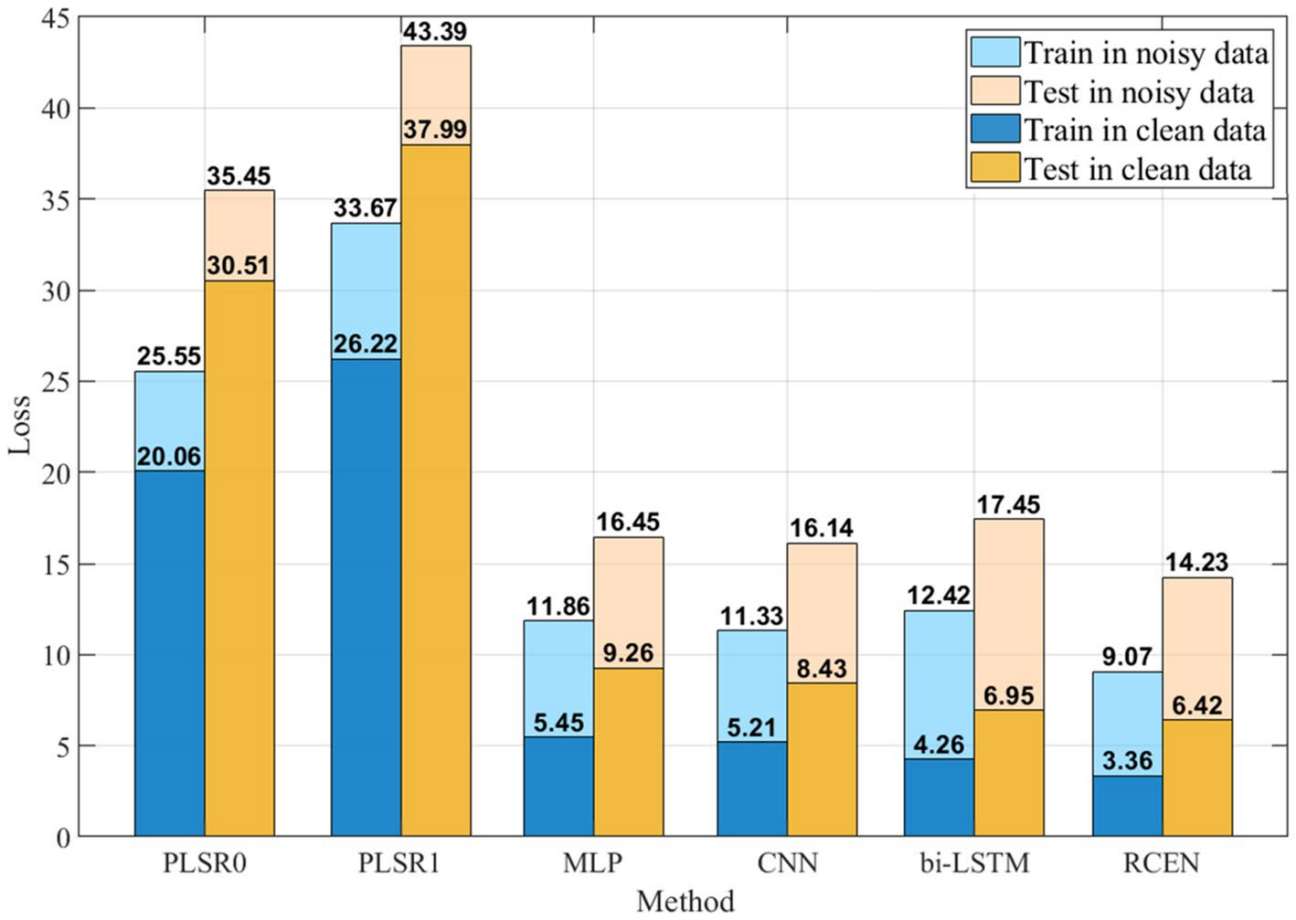
Estimation performance of the different methods for modeling the cable effect.

Next, we evaluate the estimation performance and generalization ability for different modeling methods: the partial least squares regression (PLSR) model in^24^, an MLP network with 6 layers, a CNN with 6 conv layers, a bi-directional LSTM network with a similar structure to^32^ and the RCEN described in section “Learning-based cable effect modeling”. All these methods use the best cable representation method for training, that is the RF representation for MLP and bi-LSTM, and the EFM representation for CNN and RCEN. We trained these models on clean measurement data and noisy measurement data, respectively. We report the training and evaluation loss for each method in Fig. 6. The neural network models are trained and tested on a 10 GB Nvidia RTX 3080 GPU. All the models are trained for 5 times, and we choose the best model in the entire training process of each method.

Here, we note that the PLSR0 represents the model in^24^ with 32 selected feature points, and PLSR1 represents the PLSR model with 10 configured feature points. In addition, all the other methods use the 10 configured feature points. The average computing time of PLSR0 and PLSR method is less than 200 $\mathrm{\mu s}$, the average computing time of the MLP, CNN, bi-LSTM and RCEN method is about 1 ms, 5 ms, 18 ms, and 34 ms, respectively. As can be seen, the PLSR method has the worst performance, since the PLSR model is a local model and cannot explain the nonlinear dynamics of the cable effect. Hence, all the learning-based methods show a significant performance improvement compared with the PLSR model. The MLP network is less accurate but has the shortest training and computing time. The CNN performs relatively better than MLP. The bi-LSTM network has promising performance with clean measurement data, and meanwhile has the largest performance decay with noisy data. Our RCEN achieves the best performance on both the training set and test set regardless of whether noise is applied. This indicates that the proposed method can have robust generalization ability for modeling the cable effect under different measurement conditions.

To better evaluate the performance of our method, we demonstrate the performance of the RCEN trained under different measurement noise in Table 2. The noise applied to the measured positions of feature points is white Gaussian noise, and the velocities of feature points are calculated using these noisy positions. We also compute the corresponding signal-to-noise ratio (SNR) of the training data to reflect and evaluate the measurement quality.

**Table 2:** Estimation performance of the RCEN under different measurement noises.

Noise	SNR (dB)	Performance	
		Train	Test
$\sigma = 0.0{\text{ mm}}$	Inf	3.3643	6.4203
$\sigma = 0.1{\text{ mm}}$	72.74	3.5029	6.9871
$\sigma = 0.2{\text{ mm}}$	66.72	4.2574	7.4179
$\sigma = 0.333{\text{ mm}}$	62.28	4.4588	8.2097
$\sigma = 0.5{\text{ mm}}$	58.76	5.6446	9.1494
$\sigma = 1.0{\text{ mm}}$	52.74	5.9922	10.9922
$\sigma = 2.0{\text{ mm}}$	46.72	8.1113	13.1932
$\sigma = 5.0{\text{ mm}}$	38.76	12.5032	16.9725
$\sigma = 10.0{\text{ mm}}$	32.74	15.1758	20.2373

As shown in Table 2, our method can have satisfactory modeling accuracy under different measurement noise. For the measurement signal with a SNR larger than 50 dB, our method has a promising and stable estimation ability. Even though the standard deviation of the measurement noise in positions is 10.0 mm, our method can give a relatively accurate estimation of cable effect (which is still better than the PLSR model trained with clean data). We have also conducted ablation studies to study the model ability with different network parameters. We test the RCEN network with different layers (numbers and filters). The results show that the performance improvement is not sensitive to the selection of layers when the residual learning framework exists.

### Experimental setup

**Figure 7:**
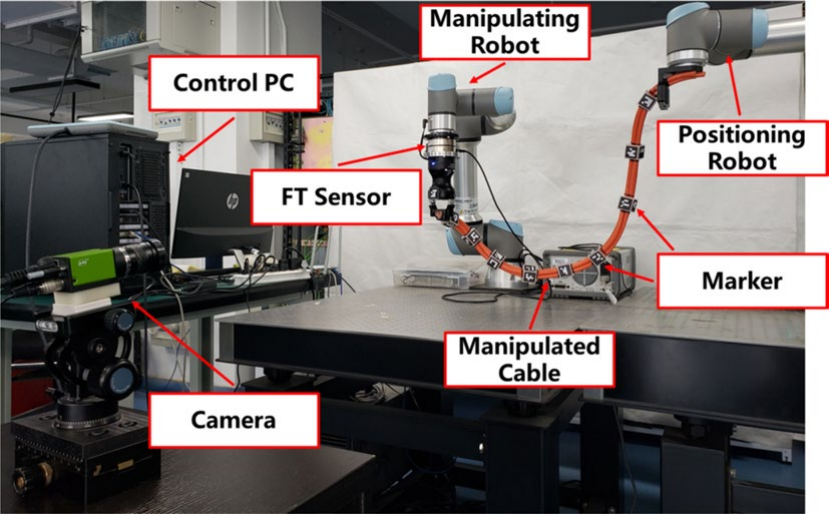
The illustration of our experimental system.

The experimental system is as shown in Fig. 7. To generate diversified training data, we use two robots to perform cable manipulation. One robot grips the cable and executes the manipulation trajectories; we call this robot the manipulating robot (a UR5e robot here). The other robot fixes one side of cable to obtain different deformation states; we call this robot the positioning robot (a UR10 robot here).

We conducted the experiment with the following process with a cyclic sequence of actions to show the repeatability and reliability of the experiments. Step 1: Before performing every manipulation trajectory, we randomly generate an initialized position of the positioning robot. Step 2: After the positioning robot achieves the initialized position, it stops running during the subsequent manipulation. Step 3: Then the manipulating robot executes the given manipulation trajectory, and we record the data for verifying our method. The cable used in the experiment is a bundle of 4 industrial cables with about 1.1 m long, and its total mass is 1.5 kg. A Basler industrial camera is utilized to capture the images at 30 Hz, and Aruco markers^36^ are used for detecting and estimating the states of the feature points. We use the same feature points configuration method as in the simulation. The center of cube is the corresponding feature point. The gripper is a Robotiq Hand-E Gripper, and the force/torque sensor is an SRI six-axis force sensor.

We randomly generate and execute 1000 experimental manipulations, with about 300 k data collected. The data is also split into training and validation with an 80–20 split. We use the method described in section “Application details” to obtain the missing measurement data. The average absolute force and torque in experiments is 20 N and 1.6 Nm, respectively. We test the same methods compared in the simulations, and Table 3 summarizes the training and evaluation loss for each method. As we can see, these methods have a similar performance on experimental data similar to that of noisy data in simulations. By comparing the quantitative results between different methods in Table 3, we reach the following conclusion: Our cable representation and modeling method can achieve the accurate estimation of the cable effect caused by manipulation. This shows that we can use the proposed method to compensate the manipulation effect of heavy cable with comparable performance of nominal no-load robot system.

**Table 3:** Estimation performance of different methods.

Modeling method	Model performance	
	Train	Test
PLSR	35.1249	46.6628
MLP	14.6217	20.9916
CNN	14.1071	19.0665
bi-LSTM	15.0160	18.5227
RCEN	13.3758	16.4741

**Figure 8:**
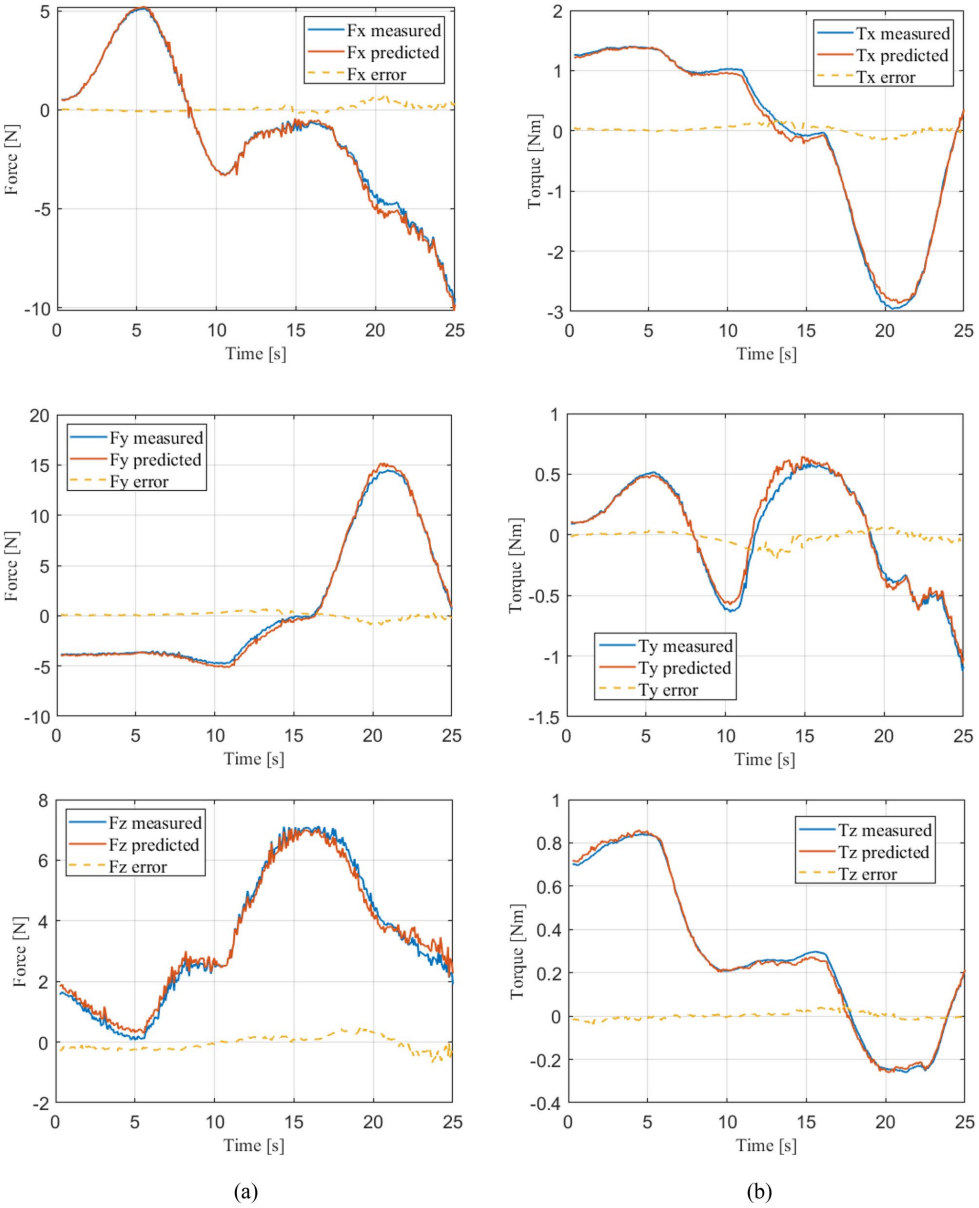
The estimation performance of our method in a real manipulation task.

The estimation performance of our method is illustrated in Fig. 8 showing the measured, predicted and their corresponding error cable effect in one experimental manipulation task. The measurement and estimation process runs with a real-time frequency of 20 Hz, and the average manipulation speed is about 0.25 m/s. Figure 8a shows the estimation performance for coupling forces and Fig. 8b shows the estimation performance for coupling torques. As we can see, the averages for predictive error forces and error torques are respectively 0.503 N and 0.087 Nm, the maximum error of predictive forces and torques are respectively 1.272 N and 0.284 Nm, which indicates over 94% of the average coupling effect can be predicted real-time in our method. For further explanation, we define the following error ratio to evaluate the prediction accuracy

6 $${\mathbf{er}}_{ratio} (t) = [\frac{{\left\| {{\mathbf{F}}_{error} (t)} \right\|_{2} }}{{\left\| {{\mathbf{F}}_{real} (t)} \right\|_{2} }},\frac{{\left\| {{\mathbf{T}}_{error} (t)} \right\|_{2} }}{{\left\| {{\mathbf{T}}_{real} (t)} \right\|_{2} }}]$$

The average error ratio in the manipulation is [5.2%, 9.1%], this accurate prediction allows for precise decoupling the effect caused by manipulated cable and serving as the modeled part in controller design, as illustrated by Figs. 1 and 10. These results shows that the proposed method has a promising performance for estimating the real-time cable effect in the robotic manipulation.

**Figure 9:**
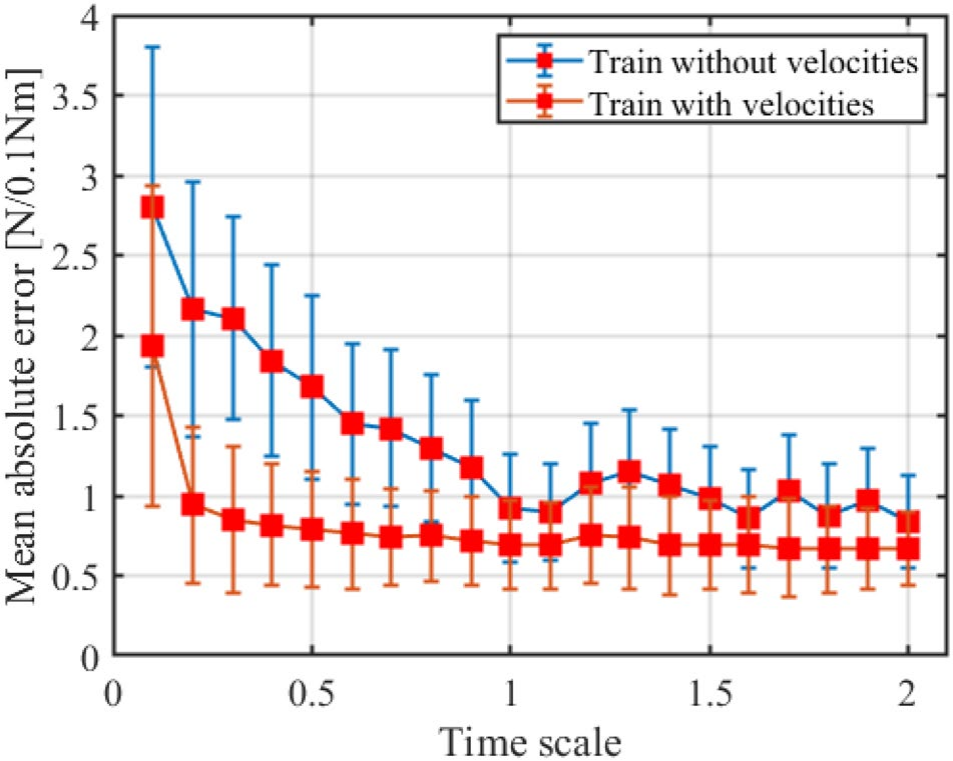
Estimation performance with the changing manipulation speed.

In our system, the velocities of feature points cannot be measured directly. The velocities of feature points are calculated using the positions, which will unavoidably amplify the measurement noise. And intuitively, the manipulation speed will affect the model performance. Now, we show the influence of the velocity features in modeling the cable effect and the effectiveness of our method. For quantitative evaluation, we test our model on a dataset with different manipulation velocities, and the result is shown in Fig. 9. Here, we use the time scale to describe the manipulation velocity and the mean absolute error with standard deviation to evaluate the performance of our method. The time scale is calculated based on the reference duration $T_{0}$ as $T_{s} \, = \, T_{m} /T_{0}$, where $T_{m}$ is the manipulation duration and $T_{0}$ is the inflexion of performance curve. A larger $T_{s}$ means a lower manipulation velocity.

As shown in Fig. 9, the model has a relatively stable performance when the manipulation runs at low speed. And the model performance will rapidly decrease when the manipulation runs faster than a threshold speed (about 0.1 m/s in our case). The result also proves that our approach can achieve better generalization in a large manipulation speed. We can also see that the estimation error sharply increases when $T_{s} = 0.1$, that is because the images captured by our camera under this consideration are rather blurred and many cable features cannot be recognized, which is one limit of our system.

By summarizing these experimental results, we can see that when the manipulation runs at speed lower than 0.25 m/s (which is a common value in application), the averages for predictive error forces and error torques are respectively 0.503 N and 0.087 Nm, the maximum error of predictive forces and torques are respectively 1.272 N and 0.284 Nm, and the average error ratio in the manipulation is [5.2%, 9.1%]. And for the manipulation speed covering 1.0 m/s (which is the limit of our system), the averages for predictive error forces and error torques are respectively 1.257 N and 0.246 Nm, the maximum error of predictive forces and torques are respectively 3.502 N and 0.425 Nm, and the average error ratio in the manipulation is [12.6%, 14.7%]. Generally, we can use the proposed cable effect model to obtain the estimation of the coupling forces and torques in the manipulation task with over 85% accuracy. We should note that the method of modeling cable effect should be used to fit different application demands, simplified method such as the MLP network and the features with RF and no velocities can be applied to meet the actual requirements of tasks. Next, we show the controller performance using the proposed method as one application of our cable effect model in cable manipulation.

### Controller performance

**Figure 10:**
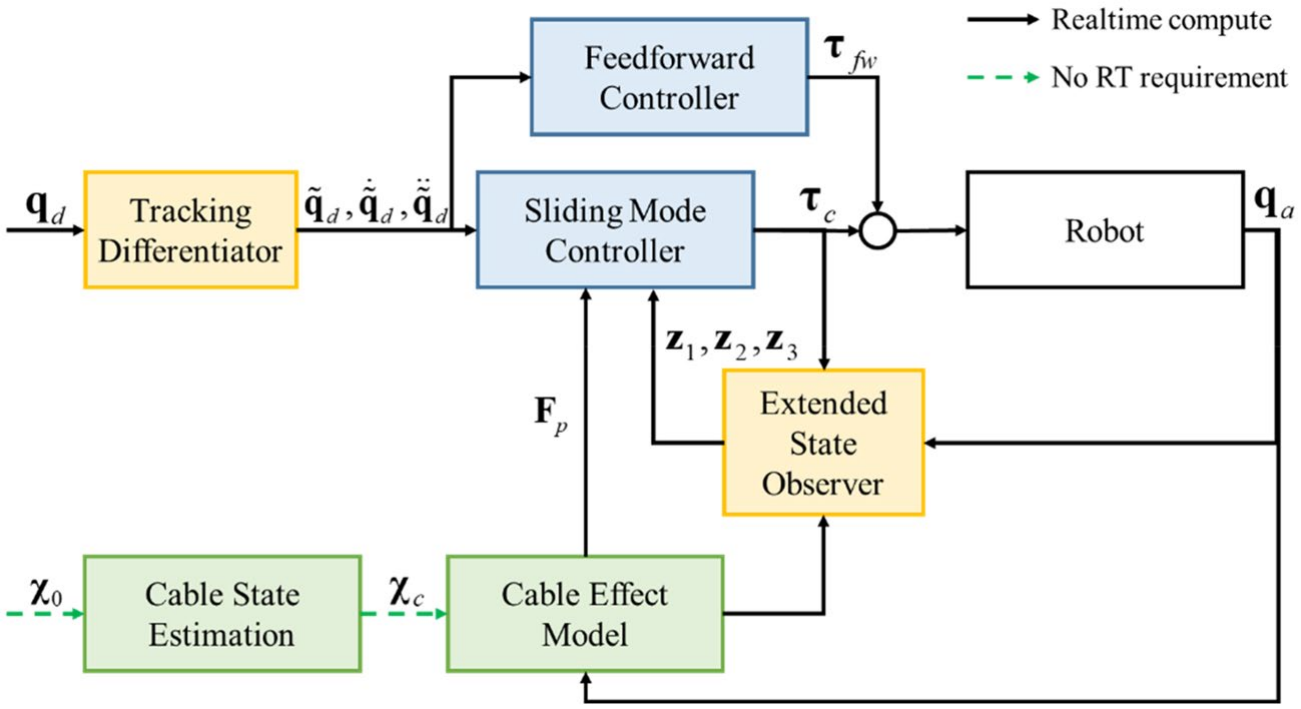
Control structure of the cable model based DRSMC framework. ${{\varvec{q}}}_{d}$ is the given trajectories of desired positions; ${\stackrel{\sim }{{\varvec{q}}}}_{d}, {\dot{\stackrel{\sim }{{\varvec{q}}}}}_{d}, {\ddot{\stackrel{\sim }{{\varvec{q}}}}}_{d}$ are the actual reference trajectories generated using the tracking differentiator; ${{\varvec{q}}}_{a}, {\dot{{\varvec{q}}}}_{a}$ are the joint angles and joint velocities of robot, respectively; ${{\varvec{z}}}_{1}, {{\varvec{z}}}_{2}, {{\varvec{z}}}_{3}$ are the augmented system states generated using the extended state observer^25^; ${{\varvec{\tau}}}_{c}$ is the feedback control torque; ${{\varvec{\tau}}}_{fw}$ is the feedforward control torque; ${{\varvec{\chi}}}_{0}$ is the measurement states of the cable and ${{\varvec{\chi}}}_{c}$ is the generated cable representation for the cable effect model.


We integrate our cable effect model in an effective and practical control framework (disturbance rejection sliding mode control, DRSMC^37^) for accomplishing the robotic manipulation of heavy cables. The DRCMS method uses the ADRC methodology to improve the robustness and accuracy of a traditional SMC controller, which provides a practical and effective trajectory tracking control framework with a strong disturbance rejection ability for robots. The cable effect model is used as the asynchronous modeled part in DRSMC framework, the block diagram of the entire control strategy is shown in Fig. 10. Algorithm 2 summarizes the control framework of the algorithm in pseudocode, detailed definitions can be seen in our preliminary study presented in^24,37^.

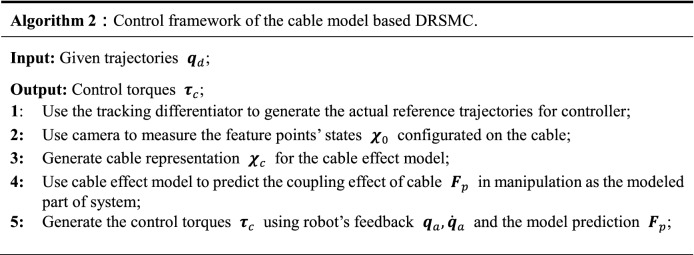


**Figure 11:**
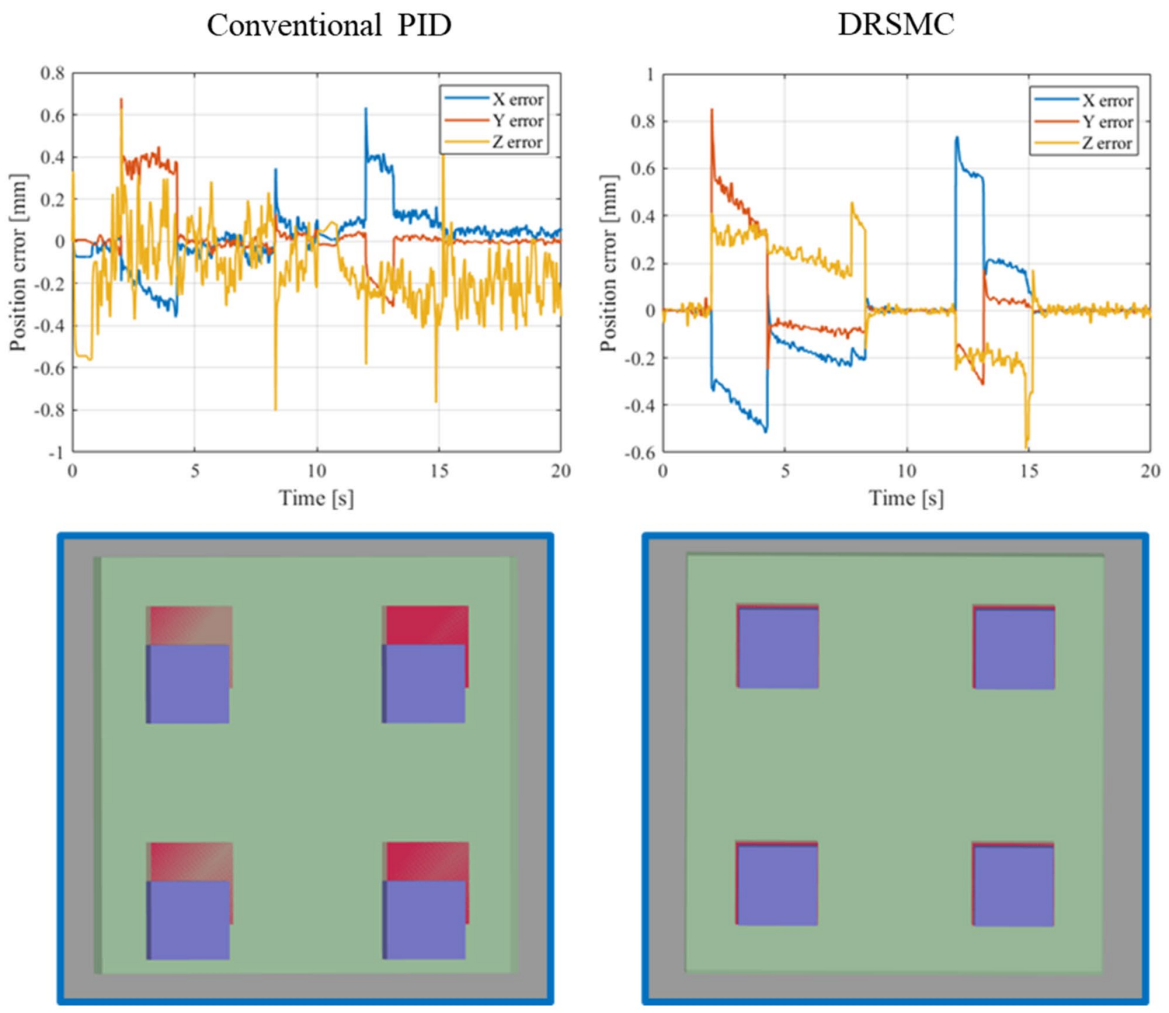
The tracking errors of manipulating point (top row) and the stable states after positioning (bottom row). The left column shows the performance using the conventional robot controller and the right column shows the performance under our method.

The performance of the controller architecture was assessed with the simulation tests described below. The simulation environment is the same robot-cable system. The cable manipulation task is to control the end of cable to reach three desired positions. The goal of the task is to reduce the positioning error of these desired positions as much as possible in order to achieve a satisfactory initial state for the subsequent insertion manipulation. The robot moves with an average speed of 0.8 m/s in Cartesian space. The standard deviation of measurement noise is 5 mm, the measuring frequency of each feature point is 10 Hz, and we randomly ignore some feature points in the measurement to simulate the real measurement condition. The cable effect model is trained using the method detailed in the above section. We first use a conventional PID controller to control the robot as commonly used in industrial applications. Then, we use our DRSMC method to complete the same robot trajectory. Both of these controllers work at the frequency of 1 kHz, and the control parameters are tuned using the same method as described in^37^. Both the PID controller and the DRSMC controller can achieve stable positioning errors of less than 0.01 mm when no payload is applied. The tracking errors of these controllers are illustrated in Fig. 11.

As seen in the top row of Fig. 11, the stable positioning errors increase to 0.3 mm under the conventional robot controller which is over 10 times compared with the no-load nominal system. This result reveals that the cable effect should be considered if high precision is needed in manipulation such as positioning or mounting in an industrial application. We can also see that using our method, the stable accuracy of manipulation can be maintained close to the nominal system that we pre-designed, which has the positioning accuracy better than 0.01 mm. In addition, the dynamic performance such as the settling times and overshoots of the system under our method are also greatly less than those of the conventional controller (as seen when *t* is near 2 s, 4.3 s, 12.5 s and 15.0 s, where the reference trajectories undergo unsmooth changes), these results indicate the promising application of robotic manipulation of a heavy cable object. The bottom row in Fig. 11 is the top view of the plug. The pins of plug are the blue parts in the figures, the socket is the green part and the holes are red parts. The width of each pin is 1.13 mm and the width of the hole in socket is 1.18 mm, which are the same objects in our future insertion manipulation task. As we can see, after the positioning manipulation, conventional controller causes relatively large errors and these errors can be greatly reduced using our method. To accomplish the following insertion manipulation, complex algorithm needs to be designed after the positioning manipulation using conventional controller. Correspondingly, simple method such as conventional impedance control can be used when the positioning manipulation finished with the errors shown in our method. The positioning errors can hardly be measured and compensated after this positioning manipulation, which leads the initial states are critical for the subsequent insertion manipulation. These results indicate that our method can greatly reduce the difficulty and increase success rate of the challenging cable insertion task.

## Conclusions

We developed a practical and accurate method for modeling the coupling effect caused by manipulating heavy cable objects. We proposed an effective and concise feature design methodology for cable representation for training the cable effect model. Practical problems on account of the engineering background such as the measurement limits and time efficiency are considered in our method for real applications. We also demonstrate the influencing factors of our method and how the performance can be improved. In addition, our approach can be effective even though the manipulation has a high speed. We evaluated the proposed approach in both the simulation environment and real-world experiments. The results showed that our method using the RCEN can obtain an estimation of the coupling forces and torques with over 85% accuracy, has reduced prediction errors by an average of 67% compared to an existing baseline model. Furthermore, we integrate our cable effect model into the DRSMC control scheme and demonstrate it satisfies the robotic manipulation of heavy cables, which can realize the cable positioning errors less than 0.01 mm compared with 0.3 mm that using the conventional method. These results show that our method can solve the problem caused by manipulating cables such as coupling dynamics and control degeneration.

The presented study has also some limitations. First, Aruco markers are used in the experiment, and the fixture of markers will partly influence the cable dynamics. Additionally, we assume that knotting and tensioning of the cable does not occur during the task while ignoring the coupling effect caused by cable twisting. These simplifications are deemed reasonable according to the manipulation tasks and the given results, but they should be considered in real-life applications, especially when the cable is less stiff.

In the future, we will use the proposed method to achieve a heavy cable insertion manipulation task. We also hope to perform our method using images of the cable without setting any markers and extend this method with more conditions considered, such as knotting and tensioning.

## Supplementary Information

Supplementary Legends.

Supplementary Video 1.

## Data Availability

The video for the paper is provided as the supplement material.
