# Supplementary material for: Learning-based cable coupling effect modeling for robotic manipulation of heavy industrial cables
Source: Sci Rep. 2022 Apr 11;12:6036. doi: 10.1038/s41598-022-09643-6 (PMC9001755; doi:10.1038/s41598-022-09643-6)
Supplement: Supplementary file 1 — Supplementary Legends. [file 41598_2022_9643_MOESM1_ESM.doc]

**Supplementary Information for:**

**Learning-Based Cable Coupling Effect Modeling for Robotic Manipulation of Heavy Industrial Cables**

Fangli Mou^1*^, Bin Wang^1^, Dan Wu^1^

^1^ State Key Laboratory of Tribology, Department of Mechanical Engineering, Tsinghua University, Beijing, China

* Corresponding author: Fangli Mou, E-mail: mfl18@mails.tsinghua.edu.cn.

**Supplementary Video and Video legend**

**Supplementary Video**. Introduction and demonstration for paper. The video contains the experiments and simulation to validate the proposed cable effect modeling and control methodology for robotic cable manipulation. The video first shows the existing research gap and the significance of our work. The first results demonstrate that our method can give accurate estimation of the cable effect in different simulated environments. The second experiment validates that our method is capable of modeling the accurate, generalizable, and efficient coupling effect of a real cable. The third experiment shows that our method is promising for robotic manipulation of heavy industrial cables with high precision.
